# High levels of kappa free light chain synthesis predict cognitive decline in relapsing-remitting multiple sclerosis

**DOI:** 10.3389/fimmu.2023.1106028

**Published:** 2023-01-20

**Authors:** Igal Rosenstein, Markus Axelsson, Lenka Novakova, Sofia Rasch, Kaj Blennow, Henrik Zetterberg, Jan Lycke

**Affiliations:** ^1^ Department of Clinical Neuroscience, Institute of Neuroscience and Physiology at Sahlgrenska Academy, University of Gothenburg, Gothenburg, Sweden; ^2^ Clinical Neurochemistry Laboratory, Sahlgrenska University Hospital, Mölndal, Sweden; ^3^ Department of Psychiatry and Neurochemistry, Institute of Neuroscience and Physiology, University of Gothenburg, Mölndal, Sweden; ^4^ United Kingdom (UK) Dementia Research Institute at University College London (UCL), London, United Kingdom; ^5^ Department of Neurodegenerative Disease, University College London (UCL) Queen Square Institute of Neurology, London, United Kingdom; ^6^ Hong Kong Centre for Neurodegenerative Diseases, Hong Kong SAR, China; ^7^ Wisconsin Alzheimer’s Disease Research Center, University of Wisconsin School of Medicine and Public Health, University of Wisconsin-Madison, Madison, WI, United States

**Keywords:** multiple sclerosis, kappa free light chains, cognitive impairment (CI), symbol digit modalities test (SDMT), biomarkers

## Abstract

**Background:**

Evolving evidence suggests that measurement of cerebrospinal fluid (CSF) kappa free light chain (KFLC) synthesis has high diagnostic sensitivity and specificity for multiple sclerosis (MS), but its prognostic ability is less investigated. The usefulness of KFLC in predicting cognitive impairment (CI) is still unknown.

**Methods:**

In a monocentric longitudinal retrospecitve cohort study, KFLC-index ([CSF KFLC/serum KFLC]/[CSF albumin/serum albumin]) measured by latex-enhanced immunonephelometry was prospectively determined as part of the diagnostic workup in patients with early relapsing-remitting MS (RRMS, n=77). The ability of KFLC-index to predict information processing speed (IPS) worsening as assessed with the symbol digit modalities test (SDMT) was investigated in univariable and multivariable models.

**Results:**

In patients with KFLC-index>100 (n=31), 11 subjects (35.5%) showed reduced SDMT scores by ≥8 points at follow-up (mean follow-up time 7.3 ± 2.6 years), compared with their baseline scores (p=0.01). Baseline KFLC-index>100 was strongly associated with a higher hazard of SDMT score reduction at follow-up (adjusted hazard ratio 10.5, 95% confidence interval 2.2-50.8, p=0.003; median time to SDMT reduction 7 years).

**Conclusion:**

Intrathecal KFLC synthesis has become an attractive diagnostic tool for MS. We show for the first time that in a real-world setting of early RRMS, KFLC-index predicted cognitive decline. Whether this predictive ability of KFLC-index also concerns other cognitive domains than IPS, warrants further investigations.

## Introduction

Multiple sclerosis (MS) is a chronic, inflammatory, and demyelinating disease of the central nervous system (CNS) (1). In its early phase, the disease is characterized predominantly by relapsing inflammatory activity, whereas in later stages, a chronic progressive neurodegenerative process seems to dominate (2). Although cognitive impairment (CI), often insidiously developed over time (3, 4), is one of the most disabling symptoms in progressive MS (5), it may occur early in the disease course, and even in the absence of other neurological manifestations (6, 7). One of the most frequently affected domains in MS-related CI is information processing speed (IPS) (8, 9), assessed with the single digit modalities test (SDMT) (10). CI often has significant effects on quality of life (11), and might be counteracted with effective disease modifying therapies (DMT) (12–14). It is therefore of vital importance to assess and predict the risk of CI development in the early stages of MS. Only a few studies have previously investigated the prognostic utility of fluid biomarkers and CI (15). Most of these studies suggest that cerebrospinal fluid (CSF) biomarkers, most prominently CSF neurofilament light (NfL), Tau, amyloid β42, chitinase 3-like proteins 1 and 2, and immunoglobulin (Ig) G oligoclonal bands (OCBs), may predict CI in MS, thus providing some insights into the mechanisms underlying the deterioration of specific cognitive domains (15).

Kappa free light chain (KFLC) index ([CSF KFLC/serum KFLC]/[CSF albumin/serum albumin]), a marker for intrathecal production of free kappa chains, has been increasingly recognized in recent years for its diagnostic (16–20) and prognostic (21–25) potential in RRMS. Although the association between intrathecal Ig synthesis as determined by IgG-OCBs and CI has been previously studied (26, 27), the prognostic role of KFLC synthesis in relation to CI remains unknown. The purpose of this study was to investigate the ability of early intrathecal KFLC synthesis to predict future worsening of IPS in patients with onset of RRMS.

## Methods

### Patients and sampling

Patients with RRMS (28) (n=165), undergoing a routine clinical investigation at the Neurology department at the Sahlgrenska University Hospital between 2013-2018 after the first demyelinating event including determination of KFLC-index, and a minimum follow-up time of four years, were retrospectively retrieved from the Swedish MS registry (SMSreg, http://www.msreg.net) (29). Excluded from the study were patients (n=84) in which SDMT was not prospectively followed. Due to possible interference of depression with SDMT assessments, patients were screened for concurrent depression with the Montgomery-Åsberg depression rating scale (MADRS) (30) and Hospital Anxiety and Depression Scale (HADS) (31). Four patients were excluded due to coexisting depression. Disability was determined annually with Expanded Disability Status Scale (EDSS) (32). Magnetic resonance imaging (MRI) of the brain and spinal cord without and with gadolinium contrast i.v. was performed on 1.5 or 3.0 T machines, according to Swedish radiological guidelines (33). Data on T1 gadolinium-enhanced lesions and new/newly enlarging T2W-lesions at follow-up was collected. Demographic data as well as data about relapses, disability, MRIs and exposure to DMTs were retrieved from the SMSreg and patients’ electronic journals.

### Score of SDMT as study endpoint

By an experienced MS nurse, patients completed the SDMT (34) within six months from diagnosis, and thereafter, annually. Patients should be clinically stable and a new version of the SDMT was used each time in order to minimize learning bias. Reduced SDMT values at follow-up of at least eight points or more (35) compared to baseline was considered significant, provided that no recovery in SDMT scores to less than eight points compared with baseline could be seen during at least one year.

### Analyses of intrathecal immunoglobulin synthesis

Matched CSF and serum samples were obtained and consecutively analysed during the routine diagnostic work-up. Concentrations of KFLC in serum and CSF were measured using the N Latex FLC kappa kit, on an Atellica NEPH 630 instrument (Siemens), following the instructions by the manufacturers. The KFLC-index was calculated using the equation [(CSF KFLC/serum KFLC)/(CSF albumin/serum albumin)]. CSF- and serum albumin levels were analysed using the ALBT2 Reagent cassette on a cobas c module instrument (Roche). The CSF/serum albumin ratio was calculated as [CSF albumin (mg/L)/serum albumin (g/L)]. Board-certified laboratory technicians, who were blinded to the clinical status, using strict procedures for quality control and run-approval, performed the analyses. All analyses were performed at the Sahlgrenska Neurochemistry laboratory in Mölndal, Sweden.

CSF-specific IgG-OCBs were determined using an in-house IEF method on 7.7% polyacrylamide gels and subsequent silver staining. Paired patient serum and CSF samples were run on adjacent lanes, and CSF-specific IgG-OCBs were defined as extra bands in the gamma-zone, which were not present in the corresponding serum sample. For quality control, a positive CSF sample with known CSF-specific OCBs was run on each gel. A cut-off value of IgG-OCB≥2 was considered positive.

### Determination of NfL and Tau in CSF

As part of the diagnostic routine for MS investigations we also measured CSF NfL (cNfL, n=77) and CSF Tau (n=73). cNfL was analyzed using a sensitive sandwich enzyme-linked immunosorbent assay (ELISA) method (NF-light^®^ ELISA kit; UmanDiagnostics AB, Umea, Sweden; Catalog # 10-7001 CE) as previously described (36), or with an in-house ELISA method as described previously in detail (37). Comparison of the in-house ELISA and the UmanDiagnostics ELISA showed cNfL concentrations in the same range and a strong linear correlation between cNfL values (37). CSF Tau concentration (INNOTEST^®^ hTAU Ag; Product # 81572) was measured by ELISA, as previously described (38).

### Statistical analysis

Data distribution was assessed with the Shapiro-Wilks test, and presented as mean ± SD or as median and interquartile range (IQR). The Mann-Whitney U test, unpaired T test, χ^2^ test, and Fisher’s exact test were used for group comparisons as appropriate. To identify the most well-suited cut-off value for KFLC-index for prognostic purposes, we calculated the fourth quintile (KFLC-index=100.8). Correlation between biomarkers and age was determined with the spearman’s rank correlation coefficient. In accordance with our results as well as previous reports on the prognostic value of KFLC-index (21, 22), we chose thereafter to dichotomise the cohort according to the cut-off value KFLC-index>100. SDMT raw score values at baseline and follow-up stratified by KFLC-index>100 were compared with the Wilcoxon matched-pairs signed rank test. The p value threshold for multiple comparisons was set with the Holm-Šídák method. A binary endpoint variable for SDMT reduction of ≥8 points at follow-up was created, which was used for the purpose of the cox proportional hazards regression models. Cox proportional hazards regression models were performed and the adjusted hazard ratios (aHR) along with corresponding 95% confidence intervals (CI) were calculated, as well as univariable Kaplan-Meier survival analyses with corresponding logrank tests. Kaplan-Meier curves are presented to visualize the results. Possible predictors of SDMT reduction were identified as those variables that statistically significantly differed between those patients who had reduced SDMT at follow-up and those who did not, i.e., age, disease duration, baseline EDSS, baseline SDMT and brain MRI characteristics (T2-weighted [T2W] lesions). Sex, MRI Gd+ lesions, evidence of disease acitivity (EDA)-3 (clinical relapses; confirmed disability worsening within 6 months, and new T1 gadolinium-enhanced lesions/new/newly enlarging T2W lesions) during the follow-up period (39), and treatment strategy (first-line therapy from start, second-line therapy from start, and escalation from first- to second-line during follow-up) did not reach statistical significance but were included in the models as they are known potential confounders (12, 40, 41). Exposure to high-dose corticosteroids prior to LP has been shown to affect KFLC serum levels (42). We adjusted for exposure to corticosteroids within 30 days prior to LP as the influence of corticosteroids on KFLC-index cannot be ruled-out. We then tested KFLC-index as a log (2)-transformed continuous predictor variable. The same analyses utilising cox proportional hazards models were performed independently and separately for cNfL and CSF Tau. We used the same covariates as mentioned above, except exposure to corticosteroids, which is not known to influence cNfL or CSF Tau levels. We tested cNfL and CSF Tau as categorical nominal variables based on calculations of the 4^th^ quintile cut-off value and as log (2)-transformed continuous variables. The 4^th^ quintile for cNfL in our cohort was 910 ng/L whereas for CSF Tau, it was 211 ng/L. Results of the other confounding covariates are presented only for the model including KFLC-index>100 as a predictive variable, as they did not substantially differ in the other models. Due to the exploratory nature of the study, no correction for multiple comparisons in the survival analyses was made. Statistical significane was assumed at p<0.05. All statistical analyses were performed with IBM SPSS version 28.0.1.0 (Armonk, NY: IBM Corp. 2011). Figures were created in GraphPad prism version 9.1.0.

### Ethical considerations

All patients included in this study had given informed consent to be registered in the SMSreg. All individual data from the different sources were made anonymous to the authors by the replacement of the personal identity numbers by unique number codes for use in the present study. The study has been approved by the Swedish Ethical Review Agency (Dnr: 2020-06851).

## Results

The study population included 77 RRMS patients, of which 54 (70.1%) were females, with a median (IQR) age at clinical onset of 32 (27.5-43) years. IgG-OCBs≥2 were present in 71 (92.2%) patients. Raw SDMT scores at last follow-up were reduced in 20 (26%) patients. KFLC-index, cNfL, and CSF Tau were non-normally distributed as was determined by the Shapiro-Wilks test of normality, whereas SDMT values at baseline and follow-up were normally distributed. Baseline demographic and clinical variables that significantly differed between patients with and without SDMT reduction were age, disease duration, total follow-up time, baseline EDSS and SDMT raw scores, as well as the number of T2 MRI lesions (**Table 1**). Sixteen (20.8%) patients had received corticosteroids within 30 days prior to LP. None of the studied biomarkers significantly correlated with age (cNfL: p=0.06; CSF Tau: p=0.84; KFLC-index: p=0.96).

**Table 1:** Clinical and demographical characteristics of RRMS patients included in the study and dichotomized by SDMT status at follow-up.

Variable	Total RRMS patients (n=77)	SDMT not reduced (n=57)	Reduced SDMT (n=20)	p-value
Age, median (IQR)	32 (27.5–43)	32 (26.5–38.5)	41.5 (30–54)	0.04^#^
Sex (Female), n (%)	54 (70.1)	38 (66.7)	16 (80)	0.4*
Disease duration from first symptoms to last visit, y, mean ± SD	9.6 ± 5.4	8.6 ± 4.7	12.2 ± 6.3	0.02^§^
Total follow-up time from first to last visit, y, mean ± SD	7.3 ± 2.6	7 ± 2.7	8.3 ± 2	0.04^§^
EDSS at baseline, median (IQR)	2 (0.5–3)	1.5 (0–2.5)	2.5 (2–3.5)	0.02^#^
Patients converting to SPMS during follow-up, n (%)	2 (2.6)	1 (1.8)	1 (5)	0.45*
EDA-3 during follow-up, n (%)	34 (44.2)	25 (43.9)	9 (45)	0.6*
Baseline SDMT raw score, mean ± SD	55.2 ± 11.4	52.7 ± 10.3	62.4 ± 11.5	0.002^§^
Follow-up SDMT raw score, mean ± SD	53.6 ± 11	54.6 ± 10.8	50.8 ± 11.4	0.2^§^
Time from baseline SDMT to censoring, y, mean ± SD	5.8 ± 2.4	5.6 ± 2.3	6.5 ± 2.5	0.2^§^
T2 MRI lesions at baseline, n (%) *1-9* *10-20* *>20*	32 (41.6) 23 (29.9) 22 (28.6)	26 (45.6) 19 (33.3) 12 (21.1)	6 (30) 4 (20) 10 (50)	0.048*
Gd+ lesions, n (%)	20 (26)	13 (22.8)	6 (30)	0.5^#^
IgG-OCBs≥2, n (%)	71 (92.2)	51 (89.5)	20 (100)	0.2*
KFLC-index, median (IQR)	50 (16.7–150.3)	36 (16–148.2)	103 (21.3–156.6)	0.26^#^
cNfL ng/L, median (IQR)	660 (373–1445)	650 (378–1580)	790 (320–1300)	0.9^#^
CSF Tau ng/L, median (IQR)	200 (166–263.5)	204 (176–274)	195 (150–245)	0.21^#^
Corticosteroids prior to LP, n (%)	16 (20.1)	12 (21.1)	4 (20)	0.92^#^
Treatment strategy, n (%): *First-line from start* *Second-line from start* *Escalated during follow-up*	9 (11.7) 35 (45.5) 33 (42.9)	8 (14) 26 (45.6) 23 (40.4)	1 (5) 9 (45) 10 (50)	0.5*
Dominant DMT, n (%): *Dimethyl fumarate* *Teriflunomide* *Fingolimod* *Natalizumab* *Rituximab* *Cladribine* *Alemtuzumab* *AHSCT*	6 (7.8) 4 (5.2) 11 (14.3) 15 (19.5) 23 (29.9) 2 (2.6) 9 (11.7) 7 (9.1)	5 (8.8) 4 (7) 6 (10.5) 11 (19.3) 20 (35.1) 2 (3.5) 4 (7) 5 (8.8)	1 (5) 0 (0) 5 (25) 4 (20) 3 (15) 0 (0) 5 (25) 2 (10)	0.2*

RRMS, relapsing-remitting multiple sclerosis; SDMT, single digit modality test; SD, standard deviation; EDSS, expanded disability status scale; IQR, interquartile range; MRI, magnetic resonance imaging; IgG, immunoglobulin G; OCB, oligoclonal bands; KFLC, kappa free light chain; cNfL, cerebrospinal fluid neurofilament light; Gd+, gadolinium enhancing; DMT, disease modifying therapy; AHSCT, autologous hematopoietic stem cell transplantation.

Data are shown as median and interquartile range unless otherwise specified. For group comparisons, the ^#^Mann-Whitney U test, *Fisher’s exact test or chi-square test, and ^§^Unpaired T-test were applied as appropriate. Bold text indicates p values<0.05.

### High levels of baseline KFLC-index predict SDMT score reduction at follow-up

**Figure 1:**
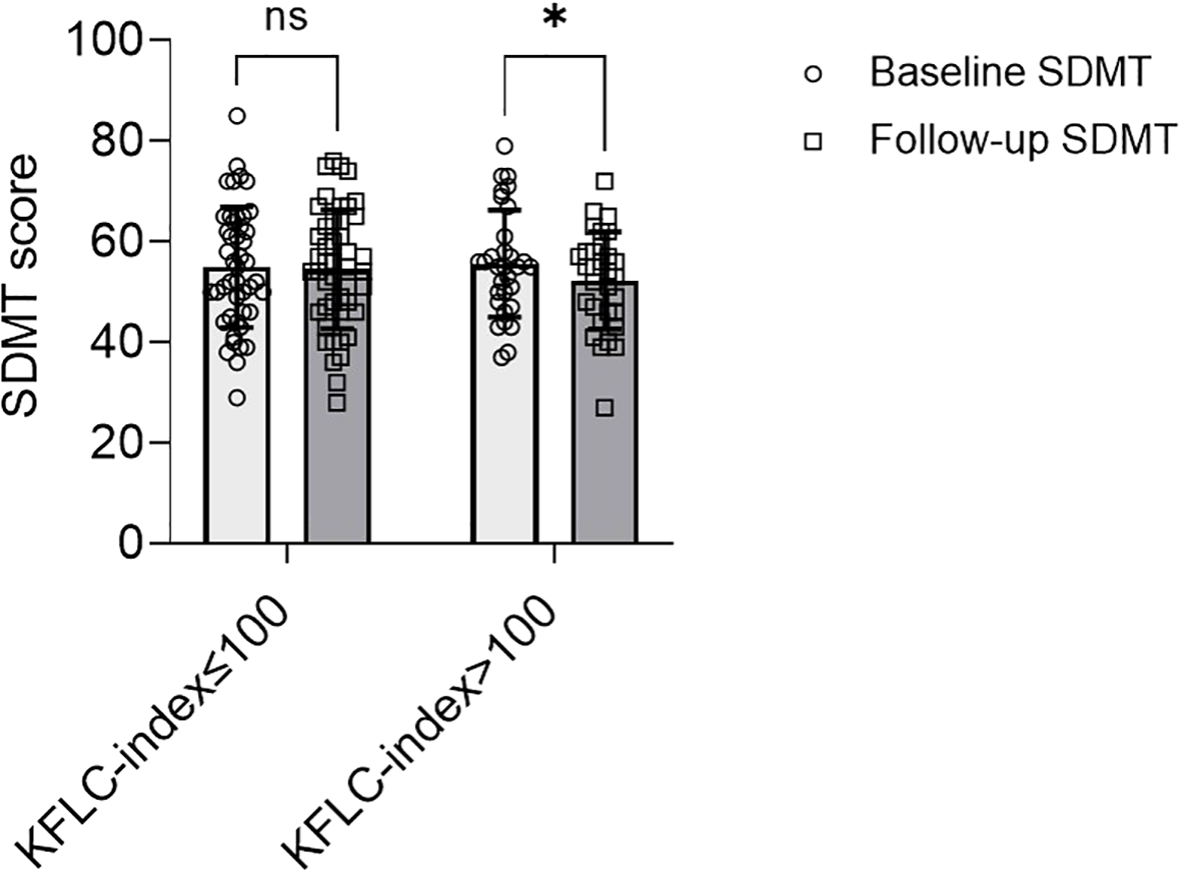
Interleaved scatter and bars plot with mean and standard deviation, showing SDMT scores at baseline compared to last follow-up in patients with relapsing-remitting multiple sclerosis stratified by KFLC-index ≤100 (n=46) vs. KFLC-index>100 (n=31). Values at baseline and follow-up were compared with the Wilcoxon matched pairs signed-rank test. Abbreviations: SDMT, single digit modalities test; KFLC, kappa free light chains. ns, non significant *p=0.01.

**Figure 2:**
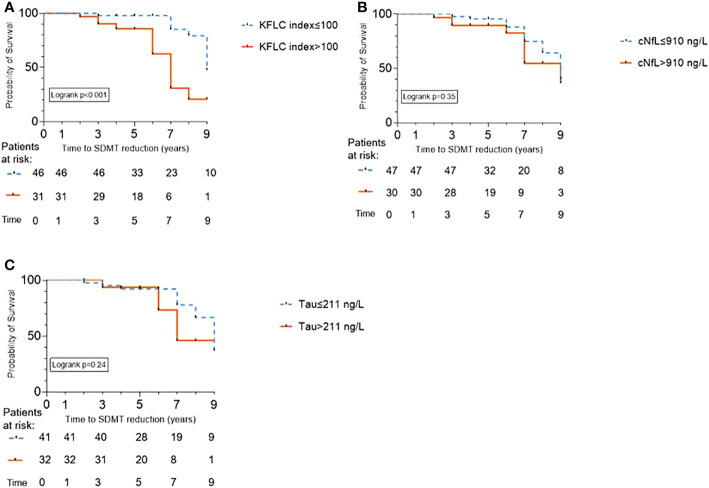
Kaplan-Meier survival curves showing time to information processing speed (IPS) worsening as predicted by cerebrospinal fluid biomarkers in patients with relapsing-remitting multiple sclerosis. IPS was measured by symbol digit modalities test (SDMT) reduction. SDMT reduction of ≥8 points compared to baseline was considered significant. Analysis of the biomarkers were performed at the time of diagnosis. A total of 77 patients were stratified according to: **(A)** The cut-off kappa free light chain (KFLC)-index>100 calculated as the 4th quintile, resulting in 31 patients in this group and 46 with KFLC-index ≤ 100; **(B)** The cut-off cerebrospinal fluid neurofilament light (cNfL) >910 ng/L calculated as the 4th quintile, resulting in 47 patients with cNfL ≤ 910 ng/L and 30 with cNfL>910 ng/L; And **(C)** The cut-off cerebrospinal fluid Tau>211 ng/L, resulting in 41 patients with Tau ≤ 211 ng/L, and 32 patients with Tau>211 ng/L.

The study population was dichotomized into patients with a high baseline KFLC-index (>100, N=31, 40%) and those with a low baseline KFLC-index (≤100, N=46, 60%). Eleven of 31 patients (35.5%) with a high baseline KFLC-index performed worse, i.e. had a sustained reduction of the SDMT score by ≥8 points at follow-up/censoring (time, mean ± SD, 6.5 ± 2.5 years) compared with baseline (p=0.01) (**Figure 1**). In patients with a low baseline KFLC-index, 9 of 46 (19.6%) had a sustained reduction of the SDMT score (p=0.83, **Figure 1**). In a multivariable analysis, KFLC-index>100 was strongly associated with a higher hazard of a sustained SDMT score reduction at follow-up (aHR 10.5, 95% CI 2.2-50.8, p=0.003) (**Figure 2A**; **Table 2**). Median time to significant sustained SDMT reduction in patients with KFLC-index>100 was 7 years compared to 9 years in patients with KFLC-index ≤ 100. The only other covariate in the model that had a significant influence on SDMT reduction was baseline SDMT score (aHR 1.1, 95% CI 1.04-1.2, p<0.001). When testing KFLC-index as a log (2)-transformed continuous variable in univariable and multivariable models, we found a significant association with higher SDMT reduction hazard (HR 1.4, 95% CI 1.06–1.8, p=0.015; and aHR 1.4, 95% CI 1.02–1.9, p=0.037 respectively).

**Table 2:** Univariable and multivariable cox proportional hazards regression models for intrathecal biomarkers measured at diagnosis as well as covariates and prediction of SDMT score reduction in RRMS patients.

Variable	Univariable model			Cox proportional hazards		
	HR	95%CI	p-value	aHR	95%CI	p-value
KFLC-index>100^a^	4.5	1.8–11.3	**<0.001**	10.5	2.2–50.8	**0.003**
Age (years)^a^	1.03	0.9–1.07	0.12	1.06	0.99–1.12	0.055
Sex (male)^a^	0.6	0.2–1.8	0.6	1.13	0.27–4.8	0.86
Steroid exposure^a^	1.4	0.5–4.1	0.6	0.6	0.15–2.4	0.45
Disease duration (years)^a^	0.96	0.9–1.06	0.4	0.9	0.8–1.05	0.23
BL EDSS^a^	1.2	0.9–1.6	0.3	1.3	0.8–1.98	0.3
BL T2W brain MRI lesions^a^ 1-9 10-20 >20	Ref. 0.4 1.2	– 0.1–1.5 0.4–3.5	– 0.2 0.7	Ref. 0.5 1.35	– 0.1–2.6 0.3–6.8	– 0.42 0.7
DMT^a^ 1^st^-line 2^nd^-line Escalation	Ref. 0.5 2.1	– 02–1.2 0.8–5.1	0.1 0.1	Ref. 0.8 1.14	– 0.04–14.9 0.7–18.7	– 0.9 0.93
Gd+ lesions^a^	2.1	0.8–5.6	0.1	1.25	0.23–6.8	0.8
BL SDMT^a^	1.05	1.02–1.09	0.004	1.1	1.05–1.2	<0.001
EDA-3^a^	1.3	0.5–3.2	0.54	1.7	0.6–5.07	0.35
KFLC-index^b^	1.4	1.06-1.8	0.015	1.4	1.02–1.9	0.037
cNfL>910 ng/L	1.5	0.6–3.8	0.35	0.56	0.12–2.7	0.5
cNfL^b^	1.3	0.9–1.7	0.17	1.1	0.8–1.6	0.6
CSF Tau>211 ng/L	1.6	0.6–4.2	0.24	0.8	0.22–2.8	0.7
CSF Tau^b^	1.5	0.63–3.7	0.35	0.6	0.17-2.03	0.4

HR, hazard ratio; aHR, adjusted hazard ratio; CI, confidence interval; KFLC, kappa free light chain; BL, baseline; EDSS, expanded disability status scale; T2W, T2 weighted; MRI, magnetic resonance imaging; DMT, disease modifying therapy; Gd+, gadolinium-enhancing; EDA, evidence of disease activity; cNfL, cerebrospinal fluid neurofilament light.

Bold text indicates p<0.05.

a Covariate results from the multivariable model including KFLC-index>100 as a predictive biomarker

b Continuous log (2)-transformed variable

### cNfL and CSF Tau did not predict SDMT worsening

The predictive value of cNfL and CSF Tau at baseline for significant reduction of the SDMT score at follow-up was assessed. Median cNfL and CSF Tau concentrations stratified by a SDMT reduction of ≥8 points at last follow-up did not significantly differ (**Table 1**). Dichotomized by 4^th^ quintile for cNfL (>910 ng/L) and CSF Tau (>211 ng/L), 30/77 (39%) and 32/73 (43.8%) respectively had increased CSF concentrations. In multivariable models, neither cNfL nor CSF Tau were significantly associated with higher hazard of SDMT reduction at follow-up (**Figures 2B** and **2C**; **Table 2**).

## Discussion

We report for the first time that high levels of KFLC-index determined as part of the diagnostic investigation may predict IPS worsening in patients with RRMS. Similar predictive value for cognitive decline was not found for cNfL or CSF Tau. It has previously been shown that the presence of IgG-OCBs is associated with a decline in IPS (26, 27), but the role of KFLC-index in predicting CI has to our knowledge never been studied before. Since high levels of KFLC-index are highly correlated with and predictive of the presence of IgG-OCBs (18), our results strengthen the notion that there is an association between early intrathecal immunoglobulin production and risk for reduced IPS. However, since more than 92% of our cohort were IgG-OCB positive, a meaningful statistical analysis could not be done. In our analysis, patients in the reduced SDMT group were older at baseline on a group level. However, their baseline SDMT scores were higher as well.

In contrast, a previous study did not show an impact from intrathecal IgM synthesis (ITMS) determined with lipid-specific oligoclonal IgM bands (LS-OCMB) on IPS worsening in RRMS (43). In fact, except for the long-term storage, assessed with the selective reminding test, no association between increased ITMS and MS-related CI was found. This is an unexpected finding since ITMS is considered a prognostic disease severity marker (44, 45). The lack of relationship found between ITMS and IPS might depend on a relatively short follow-up period of four years as well as a limited sample size (n=44) (43). Thus, further studies with larger cohorts and longer follow-up time are warranted to investigate if also other measures of early intrathecal immunoglobulin synthesis are involved in the development of CI in MS.

High levels of KFLC-index have recently shown prognostic value in terms of predicting a second demyelinating event (22), reaching EDSS 3 (23), and the need of highly efficacious DMTs (23). The cut-off value of KFLC-index>100 chosen for our analysis was used as a result of calculation of the 4^th^ quintile which has previously been used as cut-off value for prognostic purposes (22), i.e. to predict early disease activity. The authors of that study used a similar method for determination of the appropriate cut-off value. In another recent study, KFLC-index>58 was found to predict the risk of achieving EDSS≥3 and for escalating therapy to highly effective DMT (23). However, they chose to stratify their cohort according to the most discriminative KFLC-index. In both cases, as well as in the present study, it is clear that high levels of KFLC-index at diagnosis are predictive of worse prognosis. In addition, when we tested KFLC-index as a log-transformed continuous variable in univariable and multivariable models, we found that the estimated risk of SDMT reduction was approximately 40% higher with every incremental increase in log (2)-transformed KFLC-index units.

The reason why KFLC-index associates with CI is not immediately obvious. Hypothetically, high levels of KFLC-index could represent an early and more pronounced immune activation of immunoglobulin-producing B lymphocytes, thus resulting in a more severe tissue injury. The important role that B cells play in cortical grey matter injury has been recently highlighted (46). Furthermore, a recent cross-sectional proteomic analysis revealed that the B cell chemoattractant Chemokine (C-X-C motif) ligand 13 (CXCL13), could differentiate MS patients with severe CI from those with mild CI or apparently cognitively normal profile (47). Both CI and CXCL13 are associated with grey matter injury (48, 49), and several studies have previously shown an association of CXCL13 with intrathecal immunoglobulin synthesis (50, 51). Thus, a link might be made between extensive B cell activation, intrathecal KFLC synthesis, and CI. Our results support the notion that the higher the B cell activation is, the higher the risk of CI.

Mounting evidence suggests that DMTs may be effective in improving cognitive function in RRMS, and CI-related domains are now often used as outcome measures in clinical trials (12, 14). Albeit recent criticism has been raised for the use of SDMT as an outcome measure in clinical trials (52), SDMT is widely considered to be a particularly sensitive and a valid test to assess IPS in MS (53). Moreover, there is strong support for including assessments of cognition in clinical trials of different DMTs. Thus, reliable predictive fluid biomarkers such as KFLC-index may play an important role for treatment decisions as well as for assessing eligible patients for future clinical trials.

Furhtermore, KFLC-index has been recently shown to predict or be associated with early inflammatory disease activity (19, 22, 24), and in the present study we show that high levels are predictive of cognitive decline. We have previously shown that treatment with fingolimod and alemtuzumab did not influence KFLC-index levels (18). However, another study showed that patients receiving treatment with highly effective DMTs (alemtuzumab, natalizumab, rituximab/ocrelizumab, mitoxantrone) had lower intrathecal KFLC synthesis compared to patients receiving less effective therapies or treatment-naïve patients (54), although this comparison was cross-sectional. It is therefore reasonable to consider high KFLC-index levels when making treatment decisions early in the disease course.

Our study is limited by the absence of data on other cognitive domains such as short- and long-term memory, visuospatial ability, and executive function that are often affected in RRMS. Although, there is no consensus on the interpretation and comparison of serial SDMT scores in individuals as well as the usage of these scores as outcome measures, the use of ≥8 point’s reduction at last follow-up compared with baseline seems to be a strict cut-off value for assessing individual IPS worsening (35). In addition, we did not collect data on education level.

We included cNfL and CSF Tau in our study because previous studies show evidence of a prognostic value of them to anticipate cognitive decline (55–59). However, in our analysis, cNfL did not predict IPS worsening. Previous investigations show that cNfL may predict overall CI or at least worsening of some specific cognitive domains. A trend towards a negative correlation between cNfL levels and follow-up IPS was reported in 86 CIS patients with optic neuritis (55). cNfL has also been shown to negatively correlate with verbal fluency scores (56) and visuospatial memory (58). In a multivariable logistic regression analysis, an association between cNfL and IPS worsening was found (57). However, the statistical methods and endpoints of this study were notably different in comparison with our analysis, and the results of the logistic regression (odds ratio 1.06 95%CI 1.00–1.11, p=0.04) are not robust. Furthermore, several prospective (60) and retrospective (61) longitudinal studies have assessed the association of serum/plasma NfL with CI and found a negative relationship with cognitive domains, whereas others have been inconclusive (62). In a recent study, a weak but significant association was found in MS patients between CSF levels of Tau and CI including reduced IPS (59), However, they did not show an association between cNfL concentrations and CI. The statistical analyses and endpoints used in our study are different and a direct comparison is therefore difficult to be made.

In summary, our data demonstrate that high levels of KFLC-index at the diagnostic work up may predict the risk for cognitive decline in patients with RRMS. We do not find an association between CI and early neuro-axonal damage according to NfL or CSF Tau concentrations in CSF. Thus, in addition to the high diagnostic precision of KFLC-index in MS we show that it may also have prognostic value. However, further investigations are warranted to reveal if this only concerns IPS or if it also involves other domains of cognition in RRMS.

## Data availability statement

The raw data supporting the conclusions of this article will be made available by the authors, without undue reservation.

## Ethics statement

The studies involving human participants were reviewed and approved by Swedish Ethical Review Agency (Dnr: 2020-06851). Written informed consent for participation was not required for this study in accordance with the national legislation and the institutional requirements.

## Author contributions

IR and JL contributed to conception and design of the study. IR organized the database, performed the statistical analysis, and wrote the first draft of the manuscript. SR wrote sections of the manuscript. IR, MA, LN, KB, HZ, and JL had major role in data acquisition. All authors contributed to the article and approved the submitted version.
